# Comparison of Carbon Supports in Anion Exchange Membrane Fuel Cells

**DOI:** 10.3390/ma13235370

**Published:** 2020-11-26

**Authors:** Van Men Truong, Ngoc Bich Duong, Hsiharng Yang

**Affiliations:** 1 School of Engineering and Technology, Tra Vinh University, Tra Vinh City 87000, Tra Vinh Province, Vietnam; ngocbich1184@tvu.edu.vn; 2 Graduate Institute of Precision Engineering, National Chung Hsing University, 145 Xingda Road, South District, Taichung City 402, Taiwan; 3 Innovation and Development Center of Sustainable Agriculture (IDCSA), National Chung Hsing University, Taichung City 402, Taiwan

**Keywords:** anion exchange membrane fuel cell, carbon support, cathode catalyst, multiwalled carbon nanotube

## Abstract

Anion exchange membrane fuel cells (AEMFCs) are attractive alternatives to proton exchange membrane fuel cells due to their ability to employ nonprecious metals as catalysts, reducing the cost of AEMFC devices. This paper presents an experimental exploration of the carbon support material effects on AEMFC performance. The silver (Ag) nanoparticles supported on three types of carbon materials including acetylene carbon (AC), carbon black (CB), and multiwalled carbon nanotube (MWCNT)—Ag/AC, Ag/CB, and Ag/MWCNT, respectively—were prepared using the wet impregnation method. The silver loading in the catalysts was designed as 60 wt.% during the synthesizing process, which was examined using thermogravimetric analysis. The elemental composition of the prepared Ag/AC, Ag/CB, and Ag/MWCNT catalysts was confirmed using X-ray diffraction analysis. The nanoparticle size of Ag attached on carbon particles or carbon nanotubes, as observed by scanning electron microscopy (SEM), was around 50 nm. For the performance tests of a single AEMFC, the obtained results indicate that the maximum power density using Ag/MWCNT as the cathode catalyst (356.5 mW·cm^−2^) was higher than that using Ag/AC (329.3 mW·cm^−2^) and Ag/CB (256.6 mW·cm^−2^). The better cell performance obtained using a MWCNT support can be ascribed to the higher electrical conductivity and the larger electrochemical active surface area calculated from cyclic voltammetry measurements.

## 1. Introduction

**Figure 1:**
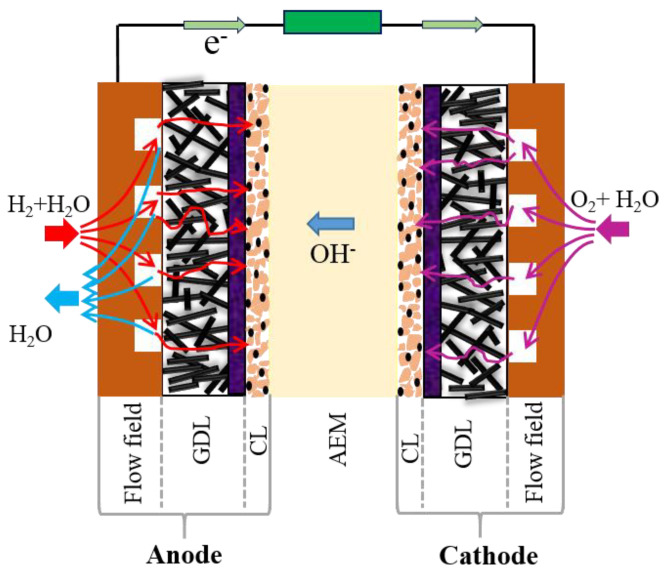
Schematic diagram of an anion exchange membrane fuel cell (AEMFC) GDL, gas diffusion layer; CL, catalyst layer.

Fuel cell technology is considered a promising alternative to power generation for the near future [1]. For fuel cell devices, low cost, durability, and reliability are the main issues that need to be addressed to commercialize this technology [2]. Among the different types of fuel cells, anion exchange membrane fuel cells (AEMFCs) have been introduced to the fuel cell research community [3] due to their advantages compared to proton exchange membrane fuel cells (PEMFCs), which are well developed. The faster kinetics of the oxygen reduction reaction (ORR) in a basic environment than in an acidic environment [4,5], allowing the use of nonprecious metals as electrode catalysts and the reduction in the corrosion problem faced by fuel cell stack hardware, which are the main drawbacks of PEMFCs, demonstrate the potential of AEMFCs as an alternative to PEMFCs. Basically, the structural design of an AEMFC stack is usually similar to that of a PEMFC stack. A typical structure of an AEMFC, consisting of anode and cathode electrodes and an anion exchange membrane (AEM) in between, is illustrated in Figure 1. During operation, H_2_ fuel and water are supplied to the anode side, and O_2_ gas and water are delivered to the cathode electrode. The electrochemical reactions occurring at the catalyst surface in an AEMFC with a direct four-electron pathway can be described as follows [6]:

At anode:

(1) $$2H_{2}+4{\mathrm{OH}}^{-}\;\to \;4H_{2}O+4e^{-};{\;E}^{0}=-0.828\;V;$$

At cathode:

(2) $$O_{2}+2H_{2}O+4e^{-}\;\to \;4{\mathrm{OH}}^{-};{\;E}^{0}=0.401\;V.$$

For most electrocatalysts employed in fuel cell devices, support materials play a critical role in determining catalytic activity and durability, as well as mass transfer and water management [3]. Carbon materials have long been used in heterogeneous electrocatalysts as a support due to their specific features such as being stable in both acidic and basic environments, satisfying most of the desirable properties required for a suitable support [7,8], and being removable from electrocatalysts by burning off, allowing an effective collection of noble catalytic metals [9]. In general, carbon materials, characterized by their high specific surface area, high electrical conductivity, and appropriate porosity, can suitably disperse metal nanoparticles, have a larger electrochemical active surface area, and have better electron transfer and water management, resulting in enhanced performance of fuel cell devices. Various carbon materials such as carbon black (CB), activated carbon, graphene (GR), and carbon nanotubes (CNTs) have been employed in different electrocatalysts [10,11,12]. Although the carbon support plays an important role in the electrocatalysts employed in fuel cell devices, limited comparisons have been conducted to evaluate and compare fuel cell performance using different carbon supports in electrocatalysts. For example, Bjorn et al. [13] experimentally investigated the influence of carbon supports such as biochar (BC), CB, GR, and CNTs on the electrocatalytic properties of Pt–Ru catalysts toward hydrogen oxidation reaction used for PEMFCs. Their results showed that the electrocatalytic activity is affected by the crystalline phase, as well as the point of zero charge. In a later study, Anuar et al. [14] conducted experiments to observe the electrocatalytic activity of iron/cobalt (FeCo) supported on various carbon materials including CB, CNTs, and reduced graphene oxide (rGO) for the ORR in an acid environment via cyclic voltammetry (CV) measurements. FeCo/rGO exhibited the highest catalytic activity according to the CV results. To the best of our knowledge, the effect of different carbon supports on the ORR in alkaline environments was only explored by Miguel and Neil [15]. In their work, Pt supported on CB, MWCNT, graphene oxide (GO), or rGO was prepared via a chemical reduction process and evaluated using the rotating ring-disc electrode technique.

In the last decade, different non-noble metals have been intensively studied as cathode catalysts in AEMFCs; among them, silver (Ag) exhibits excellent catalyst activity for the ORR in alkaline environments and relatively high stability at different working temperatures [16,17]. In alkaline media, the ORR on Ag sites can proceed via a four-electron transfer process [18,19,20] and the catalytic activity of Ag toward ORR is close to that of platinum (Pt) [21,22,23]. Therefore, following our previous work [23], we employed Ag as the metal catalyst to develop non-Pt catalysts supported on carbon for the ORR in AEMFCs. In addition, different carbon supports and metals have different interactions that significantly influence the electrochemical behavior of catalysts [13]. From our point of view, more studies on the development of non-noble metal catalysts are needed to bring AEMFC technology to the market.

This work was motivated by the need for further understanding of the effect of different carbon supports in a non-noble metal-based cathode catalyst on AEMFC performance. This is the first report on preparing a non-noble metal (i.e., Ag) supported on different types of commercial carbon materials, including acetylene carbon (AC), carbon black (CB), and multi-walled carbon nanotube (MWCNT), and evaluating their performance via an AEMFC system. Silver nanoparticles deposited on the different carbon supports were prepared using a wet impregnation method. The prepared catalysts were physically and chemically characterized. Then, the performance of the prepared catalysts was directly evaluated by integrating them into a single AEMFC. The experimental results showed that the cell performance when employing the Ag/MWCNT cathode catalyst was the highest of those tested, suggesting that MWCNT shows promise as a support material for electrocatalysts in AEMFCs in terms of improving cell performance. The findings of this work contribute to the development of non-noble metal catalysts used in AEMFCs.

## 2. Experimental Materials and Methods

### 2.1. Ag/C Catalyst Synthesis

Three types of commercial carbon materials (acetylene carbon (AC), carbon black Vulcan XC-72R (CB), and multiwalled carbon nanotube (MWCNT)) were used as catalyst supports. They were pretreated with 20% nitric acid (HNO_3_) at 120 °C in a reflux system for 2 h to create functional groups on the carbon particle surfaces [24]. Subsequently, the functionalized carbon materials were collected by filtering and washing with deionized (DI) water five times. After that, they were dried in an oven at 110 °C for 12 h.

Silver nanoparticles attached onto the carbon surfaces were synthesized using the wetness impregnation method previously reported [23]. Firstly, 185 mL of 50 mM trisodium citrate (Na_3_C_6_H_5_O_7_) was prepared and 185 mL of 10 mM silver nitrate (AgNO_3_) was added. We used Na_3_C_6_H_5_O_7_ to prevent the agglomeration of silver particles during the reduction step. Then, 251 mL of 7.4 mM sodium borohydride (NaBH_4_) was dropped slowly to the mixture under vigorous stirring using a rotary laboratory shaker. Subsequently, 200 mg of the treated carbon material was dispersed into the Ag colloid, followed by stirring for 12 h. The product was filtered and washed with DI water five times before drying in an oven at 80 °C for 12 h. Finally, the catalyst powders were collected and named 60 wt.% Ag/AC, 60 wt.% Ag/CB, or 60 wt.% Ag/MWCNT corresponding to AC, CB, or MWCNT, respectively.

### 2.2. Catalyst Characterization

Fourier-transform infrared spectroscopy (FTIR) was used to examine the carboxyl groups on the carbon surface before usage for catalyst synthesis. The composition and morphology of the as-synthesized catalysts were analyzed using high-resolution X-ray diffraction (XRD, D8 SSS, Bruker, Billerica, MA, USA), scanning electron microscopy (SEM, JSM-6700F, Tokyo, Japan), and energy-dispersive X-ray (EDX). The silver mass loadings in the catalysts were measured using thermogravimetric analysis (TGA, STA 6000 model, PerkinElmer, Waltham, MA, USA) at a heating rate of 10 °C/min from room temperature to 930 °C under a nitrogen (N_2_) environment.

The electrocatalytic activity of the prepared catalysts was characterized by cyclic voltammetry (CV) measurements. The experiments were carried out in a 1 M potassium hydroxide (KOH) solution with a three-electrode configuration using a CHI 600E workstation (CHI, Houston, TX, USA). The working electrode was prepared by coating the as-synthesized catalysts on a glassy carbon electrode (GCE). The working surface of the GCE was 0.071 cm^2^. The Ag/AgCl electrode (0.196 V vs. standard hydrogen electrode) and a Pt wire were employed as the reference and counter electrodes, respectively. The procedure of working electrode preparation was as follows: firstly, the GCE working surface was cleaned using a polishing pad and a 0.05 µm alumina slurry for 5 min, and then immediately rinsed with DI water in an ultrasonic bath for 15 min. The catalyst powder was dispersed into the DI water before adding isopropyl alcohol (IPA) and aQAPS-S_14_ ionomer (purchased from Hephas Energy Co., Ltd., Hsinchu, Taiwan). For example, the Ag/CB catalyst ink was prepared as follows: 4.17 mg of the synthesized Ag/CB powder was dispersed into 9.5 mL of DI water in an ultrasonic bath for 10 min. Subsequently, 9.5 mL of IPA and 67 µL of the ionomer were added, followed by sonicating for 1 h to produce a homogeneous solution. After that, the catalyst ink was immediately deposited on the GCE surface by drop-casting with the Ag metal loading of 35.5 µg_Ag_·cm^−2^, followed by drying at room temperature (~25 °C) for 30 min. All CV measurements were recorded at room temperature (~25 °C).

### 2.3. Fuel Cell Assembly and Testing

To evaluate the cell performance of the prepared catalysts, they were integrated into the membrane electrode assembly and tested with a single H_2_/O_2_ AEMFC. GDL-310 carbon paper with a thickness of 310 µm (Cetech Co., Ltd., Taichung, Taiwan) was used as gas diffusion layer (GDL) in both the anode and the cathode. The commercial membrane AT−1 with a thickness of 30–40 µm and aQAPS-S_14_ ionomer (2 wt.% dimethylformamide, Alfa Aesar) were purchased from Hephas Energy Co., Ltd, Hsinchu, Taiwan. Their ion exchange capacity and specific ion conductivity were about 1.0 meq·g^−1^ and 0.1 S·cm^−1^ at 60 °C, respectively. Before making membrane electrode assembly (MEA), the as-received membrane was dipped in 1 M KOH solution for 48 h at a temperature of 65 °C in to convert chloride form (Cl^−^) into the hydroxide form (OH^−^). The gas diffusion electrodes (GDEs) were prepared by coating catalyst inks on the microporous layer (MPL) surface of the GDL. The procedure was as follows: firstly, the catalyst ink was obtained by dispersing catalyst powders (Pt/C, Ag/AC, Ag/CB, or Ag/MWCNT) in the mixture of IPA and DI water as the solvent/dispersant with a volumetric ratio of 1:1 for IPA:DI water, followed by adding 25 wt.% ionomer and sonicating in an ultrasonic bath for 1 h. After that, the catalyst ink was coated on the MPL surface by hand-brushing. During this step, the GDL was placed on a hot plate with a setting temperature of 80 °C to dry catalyst layer. The catalyst loadings at the anode (40 wt.% Pt/C) and at the cathode (60 wt.% Ag/CB, 60 wt.% Ag/AC, or 60 wt.% Ag/MWCNT) were 0.8 mg·cm^−2^ and 1.0 mg·cm^−2^, respectively. Lastly, the prepared electrodes were also immersed in 1 M KOH solution to convert Cl^−^ into OH^−^ in the ionomer before assembly to form the MEA.

Before each test, the pretreated membrane was placed between the prepared anode and cathode electrodes without hot pressing to form the MEA. The active electrode area was 10.24 cm^2^. Then, the MEA was integrated into a test cell consisting of two graphite plates, two current collector plates composed of gold coated-copper, and two aluminum end plates. The triple serpentine flow channel with a rectangular cross section (width: 1 mm, height: 1 mm, and rib width: 1.5 mm) was directly machined on the graphite plate. The MEA was completely sealed by Teflon gaskets to prevent leakage of gases. The gasket thickness of 250 µm was used to provide 10–30% compression on each GDE. The cell fixture was fixed by eight bolts at a constant torque of 1.47 N·m for each bolt and mounted on a FCED-PD50 test station (Asia Pacific Fuel Cell Technologies, Ltd., Miaoli, Taiwan). During each test, the cell temperature and flow rates of humidified H_2_/O_2_ gases were set to 70 °C and 1.0/0.5 standard liter per minute (slpm), respectively. The controlled dew points of supplied H_2_ and O_2_ gases were 65 and 70 °C, respectively.

## 3. Results and Discussion

### 3.1. Catalyst Characterization

**Figure 2:**
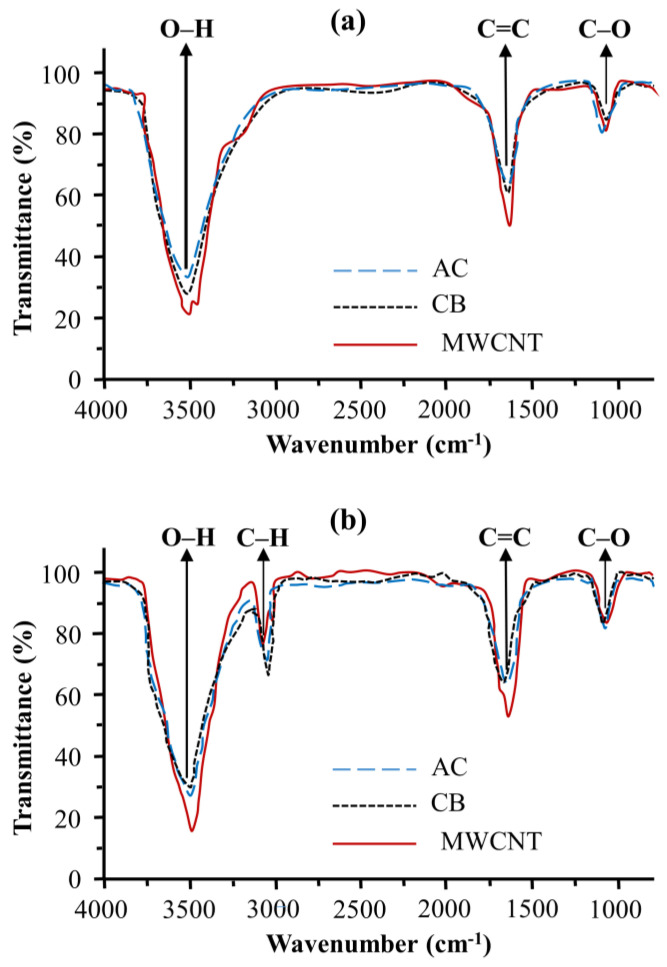
Fourier-transform infrared (FTIR) spectra of carbon materials: (**a**) before and (**b**) after treatment.

Figure 2 presents the FTIR transmission spectra of the three types of carbon materials with and without treatment with HNO_3_. The peak at around 1645 cm^−1^ could be assigned to the C=C stretching vibration of the graphite band [25]. The broad and intensive band at around 3500 cm^−1^ arose from the O–H stretching vibration from carboxyl groups (O=C–OH and C–OH). The band observed in the untreated samples could be due to surface oxidation caused by purification during the manufacturing process. The peak at about 1077 cm^−1^ could be assigned to the C–O stretching mode of the carboxylic groups [26]. In addition, the newly observed peak at 2955 cm^−1^ belonged to the asymmetric and symmetric stretching mode of carboxyl groups [27]. From FTIR analysis, we found that the carboxyl groups were generated on the carbon surface after treatment with HNO_3_. The FTIR spectra of the different carbon samples were similar, confirming the homogeneity of these samples after treatment.

**Figure 3:**
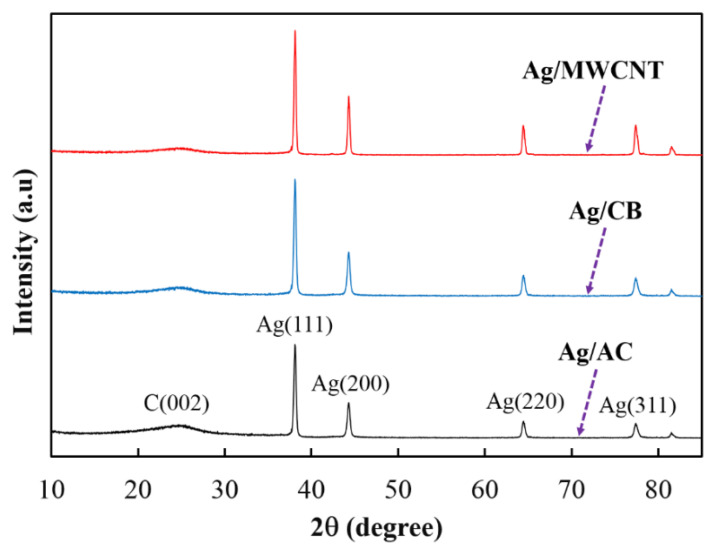
X-ray diffraction (XRD) spectrum patterns of the synthesized catalysts.

Figure 3 shows the X-ray diffraction (XRD) patterns for Ag/AB, Ag/CB, and Ag/MWCNT. The wide peak at a 2θ of about 25° was associated with the carbon (002) facet [28], while the peaks at the 2θ angles of around 38.2°, 44.3°, 64.1°, and 77.4° in the XRD patterns of these samples corresponded to the reflection of the (111), (200), (220), and (311) planes of the face-centered cubic (fcc) structure of Ag, respectively [29]. In addition, there were no obvious oxide peaks of Ag observed in the XRD patterns, indicating that the Ag nanoparticles formed in the three prepared catalysts were in metallic form. The XRD results also showed that there were only two main elements, Ag and C, observed in the analyzed samples, confirming that high-purity catalysts were obtained from the wet impregnation process.

**Figure 4:**
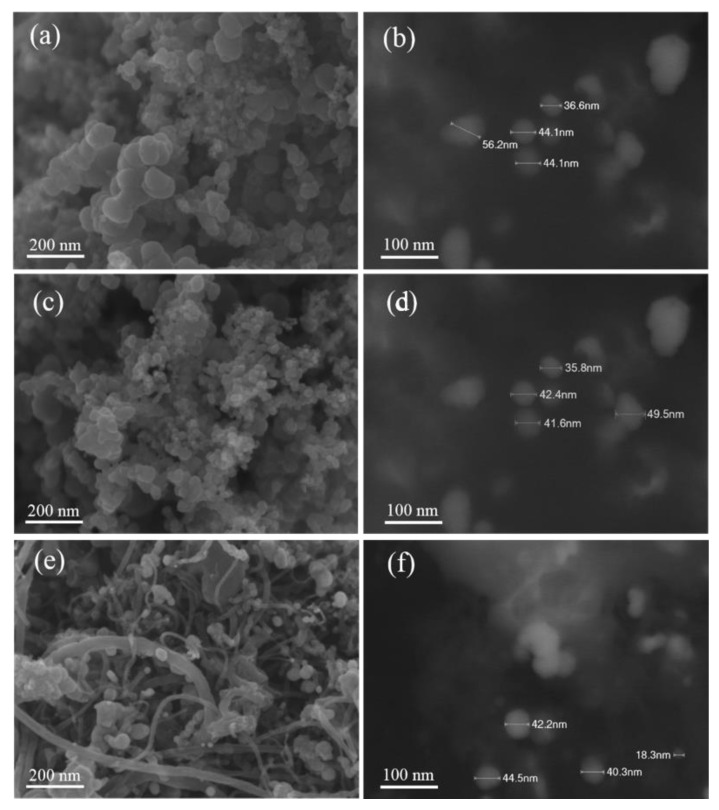
SEM images: (**a**) secondary electron image (SEI) and (**b**) composition (COMPO) mode for Ag/acetylene carbon (AC); (**c**) SEI and (**d**) COMPO mode for Ag/carbon black (CB); (**e**) SEI and (**f**) COMPO mode for Ag/multiwalled carbon nanotube (MWCNT).

**Figure 5:**
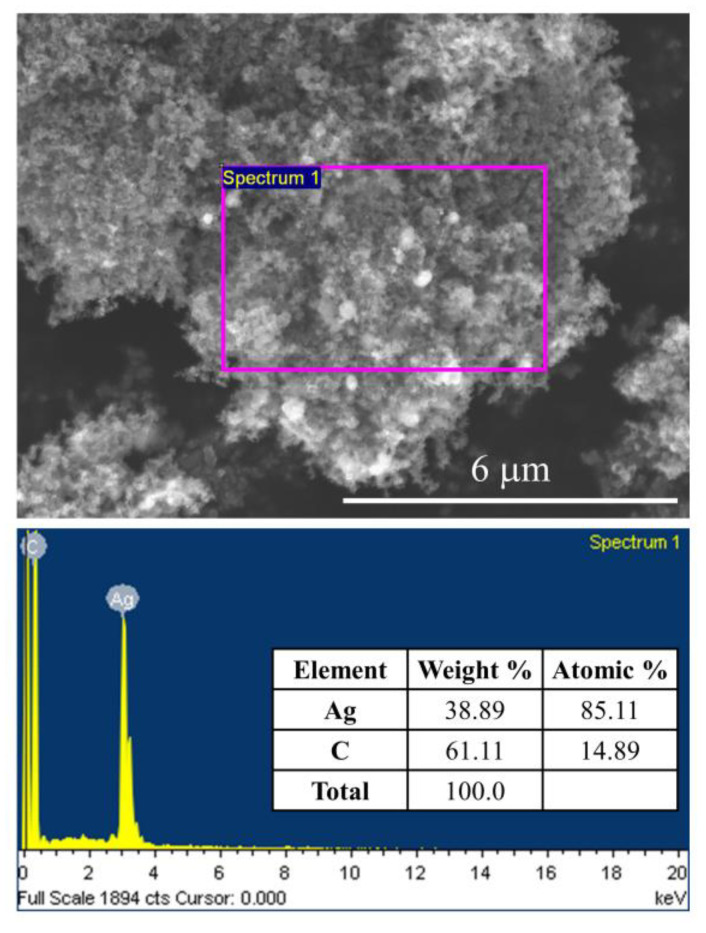
Energy-dispersive X-ray (EDX) pattern of typical Ag/CB catalyst.

The catalyst morphology was further characterized using SEM and EDX analyses. The typical SEM images of the prepared Ag/AC, Ag/CB, and Ag/MWCNT catalysts are presented in Figure 4. In the secondary electron image (SEI) mode, the surface morphology of the catalysts was observed. Although it was difficult to distinguish the carbon and silver nanoparticles in the Ag/AC (Figure 4a) and Ag/CB (Figure 4c) catalysts, the SEM image of Ag/MWCNT (Figure 4e) indicated that the silver nanoparticles were heterogeneously distributed in the catalyst. The Ag nanoparticle sizes in these catalysts were around 50 nm, and some agglomeration zones of Ag nanoparticles can be observed in Figure 4b,d,f in composition mode (COMPO mode). This could be due to the high Ag metal loading in the catalysts. We found that the shape and size of Ag nanoparticles were strongly influenced by the synthesis method and mass loading [30]. In this study, 60 wt.% Ag in the prepared catalysts was designed according to a previous report [18] stating that the Ag/C cathode catalyst with 60 wt.% Ag loading exhibited higher cell performance than those with 40 or 80 wt.% Ag loadings. Similar to the XRD results, the typical EDX pattern of the Ag/CB, as shown in Figure 5, also revealed two main elements, C and Ag, observed in the synthesized Ag/CB catalyst; their contents are reported in this figure. The measured Ag loading (61.11%) was similar to the nominal value (60%) designed in the catalyst preparation process.

**Figure 6:**
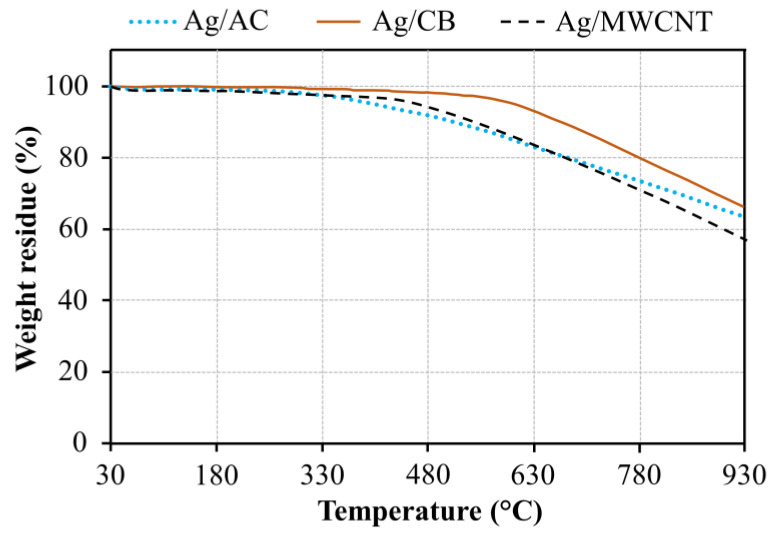
Thermogravimetric analysis (TGA) graphs of the Ag/AC, Ag/CB, and Ag/MWCNT catalysts.

The metal loadings of the prepared Ag/AC, Ag/CB, and Ag/MWCNT catalysts were further determined using TGA. The melting point of Ag nanoparticles is about 961 °C (reported by AMERICAN ELEMENT^®^, Los Angeles, CA, USA) and carbon particles completely burn at a temperature of approximately 920 °C [31]. Therefore, the maximum temperature in the TGA measurement was set to 930 °C. Figure 6 shows the thermogravimetric graphs of the catalysts from room temperature (~30 °C) to 930 °C under a N_2_ environment. The results showed that there was an initial weight reduction (~7%) from 30 to 440 °C, which could be ascribed to the loss of moisture [32]. After that, the noticeable weight loss was due to the decomposition of carbon materials. At 930 °C, the remaining weights of the analyzed samples were 63.3%, 64.1%, and 57.4% for Ag/AC, Ag/CB, and Ag/MWCNT, respectively, which could be assigned to the Ag metal. The measured Ag loadings were close to the calculated value in the synthesized process, indicating that the process was reliable. The slight difference amongst the samples could be attributed to the heterogeneous distribution of Ag nanoparticles in the synthesized catalysts. This result revealed that the influence of catalyst loading on cell performance during each testing for different MEAs was negligible.

**Figure 7:**
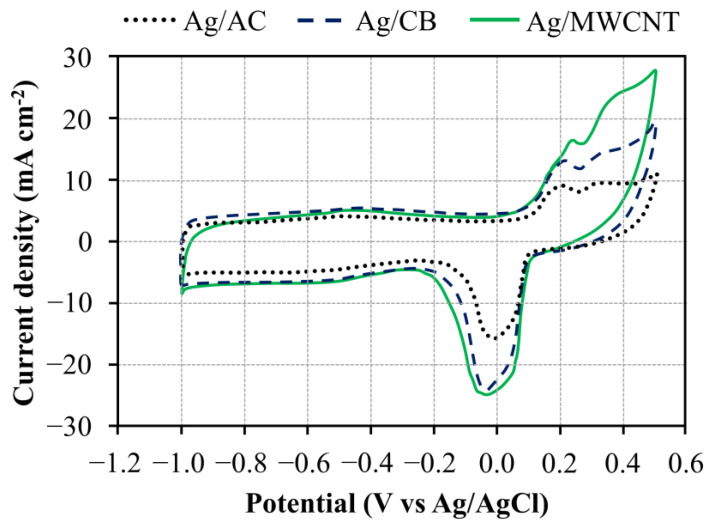
Cyclic voltammetry (CV) of different catalysts in 1 M KOH at scan rate of 100 mV·s^−1^.

The CV curves of Ag/AC, Ag/CB, and Ag/MWCNT catalysts in 1 M KOH solution at a scan rate of 100 mV·s^−1^ are shown in Figure 7. In the potential window between −1.0 and 0.5 V vs. Ag/AgCl, the anodic and cathodic peaks were observed at about 0.2 and −0.02 vs. Ag/AgCl, respectively, which are similar to those presented in the literature [33,34]. The electrochemical active surface area (EASA) is a key parameter when developing the electrodes for fuel cells [35,36]. Therefore, the EASAs of the prepared catalysts were determined from the CVs for comparison in this study. The EASAs were obtained on the basis of the oxide reduction peak of Ag(I) to Ag(0), which is the cathodic peak in the CV curves [33]. Accordingly, the estimated EASAs of Ag/AC, Ag/CB, and Ag/MWCNT were 98.4, 139.9, and 170.4 m^2^·g^−1^, respectively. Several main factors affect the EASA including metal particle size, metal loading, and interparticle distance [37]. From the above analysis, we found that the Ag nanoparticle size and its loading in the three prepared catalysts, Ag/AC, Ag/CB, and Ag/MWCNT, were not much different. Hence, the difference in the EASA of the catalysts could be attributed to the interparticle distance. In other words, the difference in EASA of these prepared catalysts was mainly associated with the specific surface area of the carbon supports [38,39], which are about 90, 215, and 245 m^2^·g^−1^ for AC, CB, and MWCNT (obtained from suppliers), respectively.

### 3.2. Fuel Cell Performance

**Figure 8:**
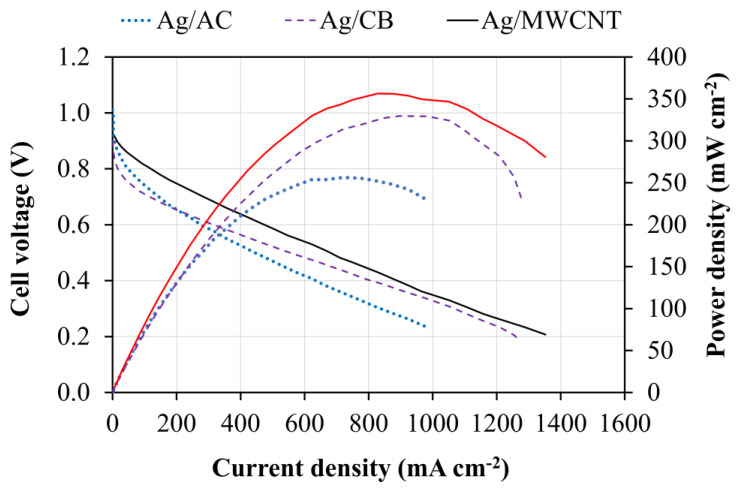
Anion exchange membrane fuel cell (AEMFC) performance using Ag/AC, Ag/CB, or Ag/MWCNT as the cathode catalyst.

For assessing the cell performance of the synthesized catalysts, different MEAs with Ag/AC, Ag/CB, or Ag/MWCNT were employed as the cathode catalysts, and commercial 40% Pt/C embedded in the anode electrode was prepared and carefully tested by a single AEMFC. This single cell was operated at a cell temperature of 70 °C under pure H_2_ and O_2_ gases with their dew points of 65 and 70 °C, respectively. The polarization and power density curves of a single AEMFC using different cathode catalysts are presented in Figure 8. Ag/MWCNT exhibited the highest cell performance. The peak power densities were 256.3, 329.6, and 356.5 mW·cm^−2^ for Ag/AC, Ag/CB, and Ag/MWCNT, respectively. The better cell performance of the cathode catalysts with different carbon supports is mainly due to the higher EASA of the catalyst, which provides more active sites for ORR, thereby improving its catalytic activity [40]. In particular, the Ag/MWCNT with the largest EASA exhibited the highest power density, followed by Ag/CB and Ag/AC. In other words, the cell performance agreed with the values of EASA calculated from the CV measurements. In addition, the higher electrical conductivity of MWCNT could be another reason for the enhanced cell performance [41]. Similar to observations in the literature, the MWCNT support is still a potential candidate to improve AEMFC performance. Although the practical applications of MWCNT are mainly hindered by its cost, by balancing the cost and cell performance improvement, MWCNT could be employed in AEMFCs. In addition, the prices of MWCNT are expected to decrease in the near future due to the improvement in MWCNT manufacturing [42,43,44].

## 4. Conclusions

The effects of various carbon supports in an Ag-based cathode catalyst on the performance of AEMFCs were experimentally studied. For physical and chemical characterization, we observed that the Ag nanoparticles in metallic form with sizes of around 50 nm were heterogeneously deposited on the three types of carbon supports, and the measured Ag loading was close to the value designed in the synthesis procedure. For single-cell evaluation, the peak power density of the single cell using the Ag/MWCNT (356.5 mW·cm^−2^) cathode catalyst was higher than that using Ag/AC and Ag/CB (256.3 and 329.6 mW·cm^−2^, respectively). The better cell performance can be ascribed to the higher EASA of the catalysts obtained from CV measurements. These results indicated that MWCNT is a promising material support for electrocatalysts applied in AEMFCs in terms of cell performance. Future examinations will focus on the interaction between Ag and MWCNT, which influences silver particle growth, using X-ray photoelectron spectroscopy or atomic force microscopy, as well as a durability evaluation of Ag/MWCNT using in situ techniques.
